# Supplementary material for: Dual PET-fMRI reveals a link between neuroinflammation, amyloid binding and compensatory task-related brain activity in Alzheimer’s disease
Source: Commun Biol. 2022 Aug 10;5:804. doi: 10.1038/s42003-022-03761-7 (PMC9365841; doi:10.1038/s42003-022-03761-7)
Supplement: Supplementary file 2 — Supplementary Information [file 42003_2022_3761_MOESM2_ESM.pdf]

TSPO PET uptake for all regions from the GLM-BOLD map

Region	t (35)	p
R superior parietal cortex (BA7)	3.662	0.006552
L Posterior Cingulate cortex (PCC)	2.814	0.031908
L ventral posterior cingulate gyrus (BA23)	2.459	0.046912
L superior parietal cortex (BA7)*	2.370	0.046912
R superior parietal cortex (BA7)*	2.183	0.057315
R Fusiform gyrus (BA37)*	1.784	0.110696
L Prefrontal gyrus (BA10)	-0.876	0.442173
R Insula	0.424	0.674108

Supplementary table 1: Group comparison in TSPO PET uptake for all regions taken from the whole brain GLM-BOLD map. Statistical significance <0.05 FDR corrected. *depicts deactivation ROIs.

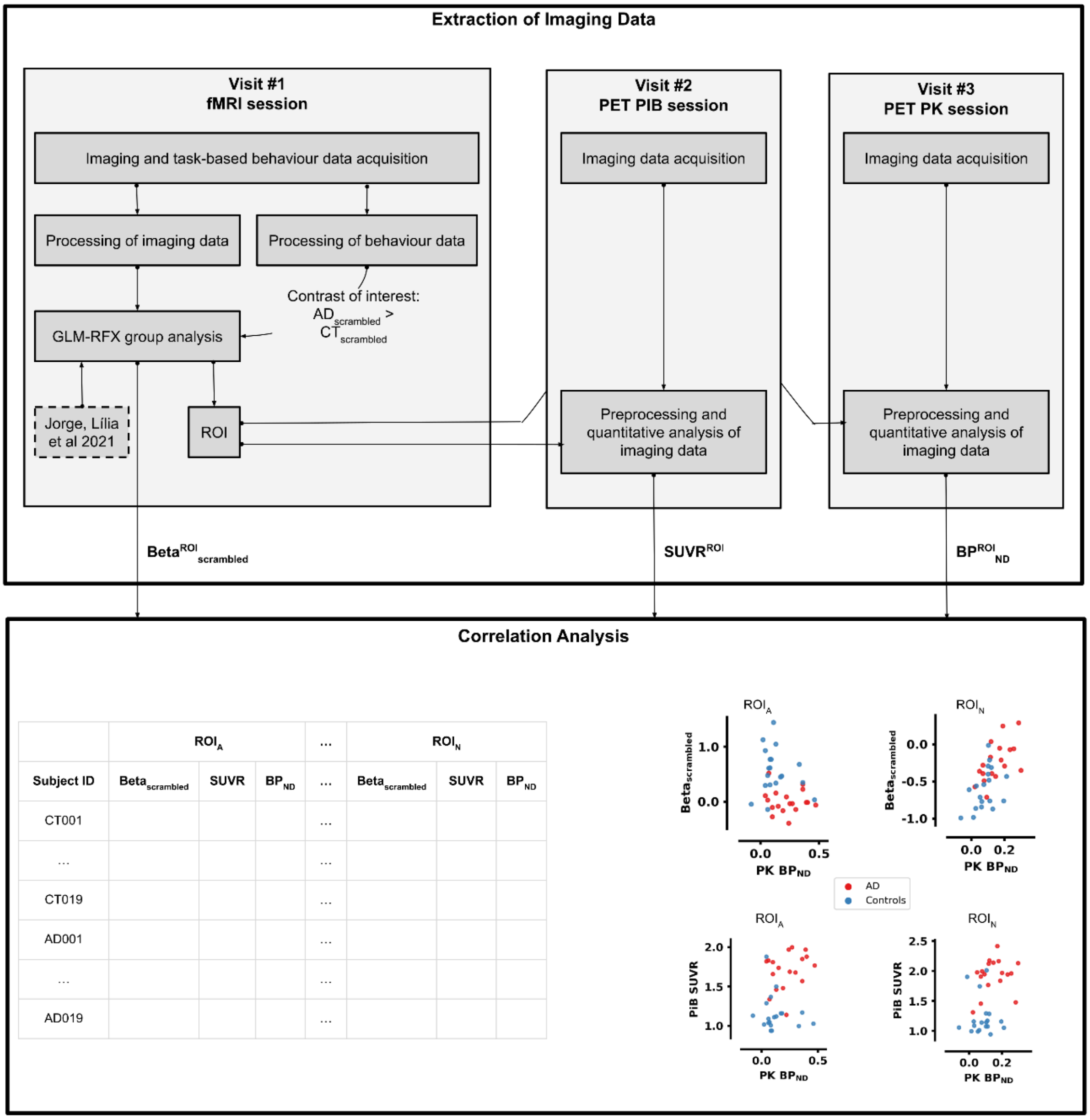

Supplementary Figure 1: Overview of the data acquisition protocol and processing pipeline.
