# Supplementary material for: Dual PET-fMRI reveals a link between neuroinflammation, amyloid binding and compensatory task-related brain activity in Alzheimer’s disease
Source: Commun Biol. 2022 Aug 10;5:804. doi: 10.1038/s42003-022-03761-7 (PMC9365841; doi:10.1038/s42003-022-03761-7)
Supplement: Supplementary file 3 — Description of Additional Supplementary Files [file 42003_2022_3761_MOESM3_ESM.pdf]

Description of Additional Supplementary Files

File name: Supplementary Data 1
Description: Supplementary Data underlying Figure 2c

File name: Supplementary Data 2
Description: Supplementary Data underlying Figure 2f
