# Supplementary material for: Dual PET-fMRI reveals a link between neuroinflammation, amyloid binding and compensatory task-related brain activity in Alzheimer’s disease
Source: Commun Biol. 2022 Aug 10;5:804. doi: 10.1038/s42003-022-03761-7 (PMC9365841; doi:10.1038/s42003-022-03761-7)
Supplement: Supplementary file 6 — Reporting Summary [file 42003_2022_3761_MOESM6_ESM.pdf]

nature portfolio

Corresponding author(s): Miguel Castelo-branco

Last updated by author(s): Jul 16, 2022

Reporting Summary

Nature Portfolio wishes to improve the reproducibility of the work that we publish. This form provides structure for consistency and transparency in reporting. For further information on Nature Portfolio policies, see our Editorial Policies and the Editorial Policy Checklist.

Statistics

For all statistical analyses, confirm that the following items are present in the figure legend, table legend, main text, or Methods section.

n/a	Confirmed	
☐	☒	The exact sample size (*n*) for each experimental group/condition, given as a discrete number and unit of measurement
☐	☒	A statement on whether measurements were taken from distinct samples or whether the same sample was measured repeatedly
☐	☒	The statistical test(s) used AND whether they are one- or two-sided<br>*Only common tests should be described solely by name; describe more complex techniques in the Methods section.*
☐	☒	A description of all covariates tested
☐	☒	A description of any assumptions or corrections, such as tests of normality and adjustment for multiple comparisons
☐	☒	A full description of the statistical parameters including central tendency (e.g. means) or other basic estimates (e.g. regression coefficient) AND variation (e.g. standard deviation) or associated estimates of uncertainty (e.g. confidence intervals)
☐	☒	For null hypothesis testing, the test statistic (e.g. *F*, *t*, *r*) with confidence intervals, effect sizes, degrees of freedom and *P* value noted<br>*Give P values as exact values whenever suitable.*
☒	☐	For Bayesian analysis, information on the choice of priors and Markov chain Monte Carlo settings
☒	☐	For hierarchical and complex designs, identification of the appropriate level for tests and full reporting of outcomes
☒	☐	Estimates of effect sizes (e.g. Cohen's *d*, Pearson's *r*), indicating how they were calculated

Our web collection on statistics for biologists contains articles on many of the points above.

Software and code

Policy information about availability of computer code

Data collection	3 Tesla Siemens Magneton Trio; Philips Gemini GXL PET/CT scanner (Philips Medical Systems, Best, the Netherlands); LOR RAMLA algorithm (Philips PET/CT Gemini GXL);Presentation 17.1 software (Neurobehavioral systems);
Data analysis	ELISAs; MATLAB R2014;SPM12; Brainvoyager QX 2.8.2; 3D Slicer 4.8.1; GingerALE v.3.0.2; SPSS v.27; Dynamic PET Kinetic Modeling and Quantification using the software validated in previous works (3), (44) and (45).

For manuscripts utilizing custom algorithms or software that are central to the research but not yet described in published literature, software must be made available to editors and reviewers. We strongly encourage code deposition in a community repository (e.g. GitHub). See the Nature Portfolio guidelines for submitting code & software for further information.

Data

Policy information about availability of data

All manuscripts must include a data availability statement. This statement should provide the following information, where applicable:

- Accession codes, unique identifiers, or web links for publicly available datasets
- A description of any restrictions on data availability
- For clinical datasets or third party data, please ensure that the statement adheres to our policy

The datasets generated during and/or analysed during the current study are available from the corresponding author on reasonable request. Supplementary Data 1-2 contain the source data underlying Fig 2c and 2f (respectively).

Field-specific reporting

Please select the one below that is the best fit for your research. If you are not sure, read the appropriate sections before making your selection.

☒ Life sciences ☐ Behavioural & social sciences ☐ Ecological, evolutionary & environmental sciences

For a reference copy of the document with all sections, see nature.com/documents/nr-reporting-summary-flat.pdf

Life sciences study design

All studies must disclose on these points even when the disclosure is negative.

Sample size	The sample size was determined by the budget and timeline of the project. The number of patients recruited was sufficient to conduct a GLM-RFX study.
Data exclusions	N/A
Replication	The description of the experimental protocol contains sufficient information in order to replicate all experimental findings.
Randomization	N/A
Blinding	N/A

Reporting for specific materials, systems and methods

We require information from authors about some types of materials, experimental systems and methods used in many studies. Here, indicate whether each material, system or method listed is relevant to your study. If you are not sure if a list item applies to your research, read the appropriate section before selecting a response.

Materials & experimental systems

n/a	Involved in the study	
☒	☐	Antibodies
☒	☐	Eukaryotic cell lines
☒	☐	Palaeontology and archaeology
☒	☐	Animals and other organisms
☐	☒	Human research participants
☒	☐	Clinical data
☒	☐	Dual use research of concern

Methods

n/a	Involved in the study	
☒	☐	ChIP-seq
☒	☐	Flow cytometry
☐	☒	MRI-based neuroimaging

Human research participants

Policy information about studies involving human research participants

Population characteristics	A total of 19 patients in the mild phase of Alzheimer's disease (MoCA= 14.26 ± 4.31, CDR=1; age: 66.11 ±7.02; sex: 10 male: 9 female; education: 8.95 ± 5.83 years, ApoE-e4 (%) = 95 %) and 19 controls (MoCA = 24.94 ± 3.62, age: 66.05 ± 6.77; gender: 10 male and 9 female; education: 10.53 ± 5.31) were enrolled in this study.
Recruitment	All 19 patients were recruited at the Neurology department of the Centro Hospitalar e Universitário. AD diagnosis was made by two experienced neurologists at the Memory Clinic of the Neurology department of CHUC. The clinical diagnosis was supported by biological biomarkers (cerebrospinal fluid - CSF and/or 11C-PiB PET SUVR). All 19 controls were recruited from the community.
Ethics oversight	Ethics Committee of Faculty of Medicine of the University of Coimbra

Note that full information on the approval of the study protocol must also be provided in the manuscript.

Magnetic resonance imaging

Experimental design

Design type	Block design
Design specifications	Each block was composed of 20 images, each one presented for ~ 800 ms followed by a ~ 200 ms of interstimulus

Design specifications	interval which represents an individual duration of 20s per block. Each block was separated by 10-s fixation with uniform grey-scale image baseline interval, representing the baseline condition.
Behavioral performance measures	Subjects performed a 1-back task during the scan session, they were instructed to press a button every time the image being presented was the same that had been presented immediately before. Each block always had four repetitions of images, that is four possible targets to which participants had to respond to, making a total of 12 chances for hits per stimulus category in each run.

Acquisition

Imaging type(s)	Structural (T1-weighted 3D anatomical MPRAGE (rapid gradient-echo)) and functional (T2*-weighted 2D echo-planar images)
Field strength	3 T
Sequence & imaging parameters	Structural: voxel size of 1.0 x 1.0 x 1.0 mm, TR of 2530 ms, TE of 3.42 ms, TI of 1100 ms, FOV of 256 x 256 mm, a flip angle of 7° and 176 slices. Functional: voxel size of 2.5 x 2.5 x 3 mm, TR of 2000 ms, TE of 30 ms, FOV of 256 x 256 mm, matrix size of 102 x 102, a flip angle of 90°, 31 slices and had 276 volumes.
Area of acquisition	Whole brain

Diffusion MRI ☐ Used ☒ Not used

Preprocessing

Preprocessing software	Brainvoyager QX 2.8.2 (BrainInnovation, Maastricht, the Netherlands).
Normalization	Brainvoyager QX 2.8.2 (BrainInnovation, Maastricht, the Netherlands).
Normalization template	Talairach reference system (TAL)
Noise and artifact removal	functional data were corrected for differences in time for each slice, were applied a filter to remove low-frequency drifts and adjusted for mean intensity. All volumes were also corrected for motion. Overall we used Brainvoyager QX 2.8.2 (BrainInnovation, Maastricht, the Netherlands) standard parameters.
Volume censoring	N/A

Statistical modeling & inference

Model type and settings	Voxel-wise random-effect general linear model (RFX-GLM)
Effect(s) tested	Statistical t maps using specific functional contrast - [scrambledAD > scrambledControls].

Specify type of analysis: ☐ Whole brain ☐ ROI-based ☒ Both

Anatomical location(s): The ROIs were extracted from the functional contrasted describe above using the RFX-GLM procedure.

Statistic type for inference (See Eklund et al. 2016)	Voxel-wise
Correction	p<0.01, BrainVoyager Cluster level threshold plugin (1000 Montecarlo simulations)

Models & analysis

n/a	Involved in the study
☒	☐ Functional and/or effective connectivity
☒	☐ Graph analysis
☒	☐ Multivariate modeling or predictive analysis
